# Systemic Lupus Erythematosus: Genomics, Mechanisms, and Therapies

**DOI:** 10.1155/2012/926931

**Published:** 2012-07-16

**Authors:** Antonio Fernández-Nebro, Sara Marsal, Winn Chatham, Anisur Rahman

**Affiliations:** ^1^Rheumatology Department, University of Málaga (UMA), Málaga, Spain; ^2^Grup de Recerca de Reumatologia, Institut de Recerca, Hospital Vall d'Hebron, Barcelona, Spain; ^3^Division of Clinical Immunology and Rheumatology, University of Alabama at Birmingham, Birmingham, AL 35226, USA; ^4^Centre for Rheumatology, Division of Medicine, University College London, London, UK

Systemic lupus erythematosus (SLE) is a complex disease caused by complex interactions between genes and the environment (sex, age, hormones, smoking, infections, drugs, and abnormalities of both the innate and adaptive immune systems). To understand the mechanisms that regulate these interactions and the processes responsible for an immune system that is increasingly autoreactive, it is essential to definitively control lupus and related disorders.

Although the prevalence of SLE among East Asians is higher than among Europeans [1], most genomes wide association studies (GWAS) have been conducted on populations of European descent. Through multinational collaborations, these studies have achieved large sample sizes and considerable statistical power. Although the sample sizes of genetic studies in East Asians are generally much smaller than those in Europeans, some have yielded new candidate loci and copy number variations [2, 3]. However, in the last 3 years, the focus has clearly switched to GWAS in an attempt to discover new risk loci that may provide unique information in complex diseases. In this special issue on SLE, we have invited H. C. Chai et al. to review the genetic factors of SLE in the Malaysian population. In their paper, these authors emphasise that most of the polymorphisms investigated did not show significant associations with susceptibility to SLE among those of Malaysian descent, except for those polymorphisms occurring in MHC genes and genes encoding TNF-*α*, IL-1*β*, IL-1RN, and IL-6. Although this could be due to smaller sample sizes, the genetic heterogeneity of SLE among different ethnicities and gene-gene or gene-environment interactions could also lead to differences in SLE susceptibility. 

It is increasingly recognised that subsets of B cells differ in function and that changes in the balance of these functionally distinct subsets may be relevant to the pathogenesis of SLE. In both a research paper and a review article, S. Koarada and colleagues describe a particular subset of B cells that do not express the Toll-like receptor homologue RP105. These RP105-negative B cells may expand in SLE and play a role in the pathogenesis of the disease. Toll-like receptors (TLRs) themselves may also be important in the pathogenesis of SLE. This applies particularly to TLR7 and TLR9, as discussed in the paper by G. Guggino et al.. TLRs are important for antimicrobial immunity, but TLRs could affect SLE through two major mechanisms. TLRs can be stimulated by exogenous antigens, such as viral RNA, which then stimulate resident immune cells [4]. Additionally, TLRs recognise endogenous self-antigens and initiate and propagate inflammation and autoimmunity [5].

TLRs are also expressed in some renal cells such as epithelial and mesangial cells. Mesangial cells have three main functions: filtration, support of glomerular capillaries, and the phagocytosis of apoptotic cells and immune complexes. An association between TLR9 and lupus nephritis has been reported in a murine lupus model and in human lupus, which indicates the possibility of crosstalk between innate immunity and autoimmunity [4, 6, 7]. Anti-dsDNA antibodies are relevant in the development of lupus nephritis, but the mechanism by which they are nephritogenic is far from clear. In this special issue on SLE, G. Seret et al. propose that some types of anti-dsDNA antibodies stimulate mesangial cells to produce cytokines, chemokines, and matrix metalloproteinases and to induce proliferation and apoptosis, matrix protein accumulation, and chromatin accumulation and immune complex formation.

Free-radical-mediated reactions are implicated in SLE. Autoimmune conditions are associated with the increased activation of immune effector cells and the production of free radical species. The generation of neoantigenic determinants by free-radical-mediated reactions increases the antigenicity of DNA, LDL, and IgG, generating ligands for which autoantibodies show higher avidity [8]. However, the potential for oxidative stress to contribute to SLE pathogenesis remains largely unexplored in humans. In the present special issue, K. J. Li and colleagues have shown that deranged cellular bioenergetics and defective redox capacity in T lymphocytes and polymorphonuclear neutrophils are responsible for cellular immune dysfunction and are related to increased oxidative stress in active SLE patients.

Patients with SLE have an increased risk of cardiovascular disease compared with the general population, leading to increased cardiovascular morbidity and mortality. In the general population, the frequency of diastolic heart dysfunction increases with age, particularly in women and in patients suffering from arterial hypertension. Isolated diastolic heart dysfunction is often demonstrated in SLE [9] and is also related to patient age. However, autoimmune diseases are known to have a role in cases of unexplained diastolic failure. In this special issue, L. M. Blasco Mata et al. present an interesting paper that aims to identify autoimmune systemic diseases in subjects with recurrent unexplained diastolic heart failure. According to these authors, up to 11% of patients with recurrent unexplained diastolic heart failure exhibit autoimmune abnormalities. 

Recent developments in several imaging techniques have improved the risk stratification of SLE patients with cardiovascular disease. In this special issue, S. C. Croca and A. Rahman have reviewed the use of various imaging techniques in the assessment of cardiovascular disease (CVD) risk in SLE. CVD is an important cause of morbidity and mortality in SLE, and the use of imaging to identify patients at risk of developing CVD before they develop symptoms is likely to become increasingly important.

*Antonio Fernández-Nebro* *Sara Marsal* *Winn Chatham* *Anisur Rahman*
